# Twofold improved tumor-to-brain contrast using a novel T1 relaxation-enhanced steady-state (T_1_RESS) MRI technique

**DOI:** 10.1126/sciadv.abd1635

**Published:** 2020-10-28

**Authors:** R. Edelman, N. Leloudas, J. Pang, J. Bailes, R. Merrell, I. Koktzoglou

**Affiliations:** 1 Radiology, NorthShore University HealthSystem, 2650 Ridge Ave., Evanston, IL 60201, USA.; 2 Northwestern Medicine, 251 E. Huron St., Chicago, IL 60611, USA.; 3 Siemens Medical Solutions USA Inc., 737 N. Michigan Ave., Chicago, IL 60611, USA.; 4 University of Chicago Pritzker School of Medicine, 924 E. 57th St., Chicago, IL 60637, USA.; 5 Neurosurgery, NorthShore University HealthSystem, 2650 Ridge Ave., Evanston, IL 60201, USA.; 6 Neurology, NorthShore University HealthSystem, 2650 Ridge Ave., Evanston, IL 60201, USA.

## Abstract

A technique that provides more accurate cancer detection would be of great value. Toward this end, we developed T1 relaxation-enhanced steady-state (T_1_RESS), a novel magnetic resonance imaging (MRI) pulse sequence that enables the flexible modulation of T1 weighting and provides the unique feature that intravascular signals can be toggled on and off in contrast-enhanced scans. T_1_RESS makes it possible to effectively use an MRI technique with improved signal-to-noise ratio efficiency for cancer imaging. In a proof-of-concept study, “dark blood” unbalanced T_1_RESS provided a twofold improvement in tumor-to-brain contrast compared with standard techniques, whereas balanced T_1_RESS greatly enhanced vascular detail. In conclusion, T_1_RESS represents a new MRI technique with substantial potential value for cancer imaging, along with a broad range of other clinical applications.

## Teaser

T_1_RESS is a novel MRI method that provides twofold improved tumor-to-brain contrast and enables modulation of vascular signal.

## INTRODUCTION

**Fig. 1:**
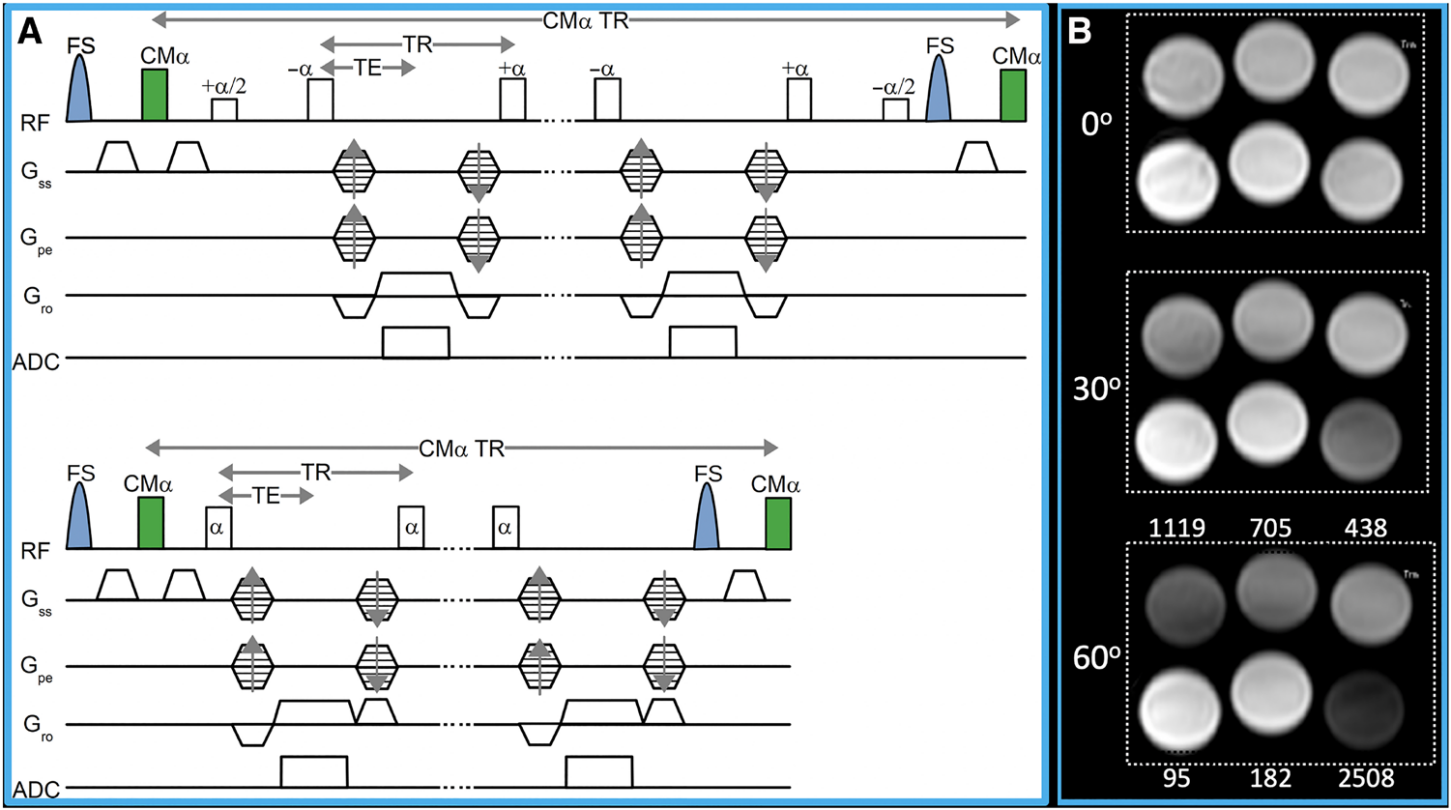
T_1_RESS pulse sequence. (**A**) Pulse sequence diagram using balanced (top) and unbalanced (bottom) steady-state readouts. α represents the imaging radio frequency (RF) pulse, and FS denotes the fat saturation RF pulse. A nonspatially selective contrast-modifying RF pulse (CMα) is applied periodically over the entire duration of the echo train to introduce an arbitrary amount of T1 weighting. For brain imaging, CMα TR ≈ 400 ms. Note that store/restore RF pulses (represented by α/2) are needed for balanced T_1_RESS, but not for the unbalanced version. ADC, analog-to-digital conversion. (**B**) Phantom consisting of serial dilutions of gadobutrol imaged with bT_1_RESS using CMα values of 0°, 30°, and 60°. T1 relaxation times in millisecond units are shown in the lowest frame. Note that there is negligible T1 contrast for a CMα flip angle of 0°, but substantial T1 contrast is apparent as the flip angle is increased to 60°.

Contrast-enhanced magnetic resonance imaging (MRI) is the cornerstone for brain tumor diagnosis (*1*). While its sensitivity for metastases is superior to that of computed tomography (CT) or positron emission tomography–CT, small lesions (<5 mm) may still be missed, which can have a major impact on prognosis and treatment planning for stereotactic radiosurgery (*2*, *3*). Triple-dose contrast administration can increase sensitivity but is seldom used because of concerns about nephrogenic systemic fibrosis (*4*). A method that could further improve the sensitivity and specificity of MRI for tumors would be of great clinical benefit. Toward this end, we have developed a new MRI pulse sequence, called T1 relaxation-enhanced steady-state (T_1_RESS), that improves the visibility of tumors in contrast-enhanced MRI by differentially suppressing the signal intensity of nonenhancing background tissues while maintaining the signal intensity of contrast-enhancing lesions. Two variants of T_1_RESS were implemented (Fig. 1A). One version, which we call “balanced” T_1_RESS (bT_1_RESS), uses a readout in which the gradient moments are fully balanced to make contrast-enhanced blood vessels appear bright. A second “unbalanced” T_1_RESS (uT_1_RESS) version renders blood vessels dark by using a steady-state readout in which the gradients are unbalanced.

## RESULTS

**Fig. 2:**
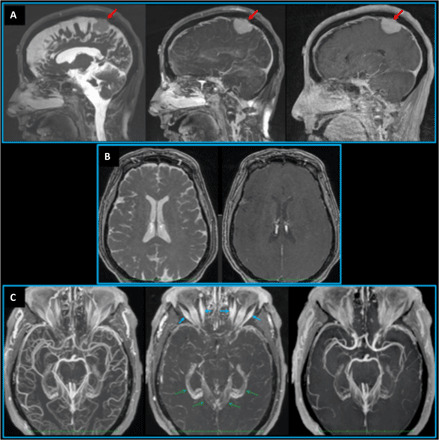
T_1_RESS sequence comparisons in different patients. (**A**) Patient with a meningioma (red arrows). Left: Unmodified three-dimensional (3D) bSSFP acquisition shows bright signal from cerebrospinal fluid and poor tissue contrast, precluding diagnostic evaluation of the tumor or blood vessels. Middle: With bT_1_RESS, cerebrospinal fluid and healthy brain tissue appear relatively dark. There is excellent visibility of the contrast-enhancing meningioma, nasal mucosa, venous sinuses, and their tributaries. Right: With 3D spoiled GRE, tumor visibility and vessel detail are inferior to bT_1_RESS. (**B**) Patient with unremarkable brain MRI. Comparison of axial multiplanar reconstructions from uT1RESS using CMα flip angles of 0° (left) and 75° (right). As with bT_1_RESS, without the effect of a CMα RF pulse, there is poor visualization of contrast-enhancing tissues (e.g., choroid plexuses). (**C**) Axial maximum intensity projections (24 mm thick) from a patient with an unremarkable brain MRI. Left: bT_1_RESS. Middle: uT_1_RESS. Right: 3D spoiled GRE. bT_1_RESS shows much more vascular detail than 3D spoiled GRE, while uT_1_RESS shows extensive suppression of intravascular signals. Because the signals from blood vessels and nonenhancing background tissues are suppressed, contrast enhancement of the extraocular muscles (blue arrows), dura, and choroid plexuses (green dashed arrows) is better shown by uT_1_RESS than by the other imaging techniques.

For both versions of T_1_RESS, the periodic application of contrast-modifying radio frequency (RF) pulses was essential for generating T1 contrast (Fig. 1B) and suppressing the signal intensity of cerebrospinal fluid (Fig. 2, A and B). bT_1_RESS outperformed spoiled gradient echo (GRE) for creating angiographic renderings in which the blood vessels appeared bright (movie S1), whereas uT_1_RESS rendered the blood vessels uniformly dark (Fig. 2C).

**Fig. 3:**
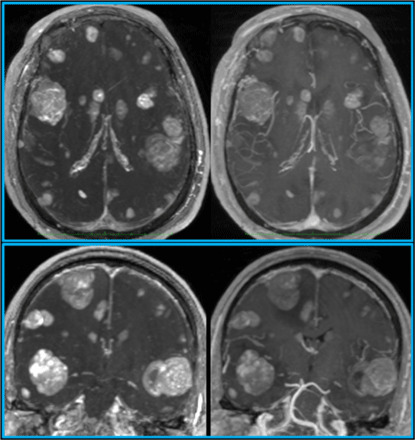
Pulse sequence comparisons in a patient with metastatic melanoma. Axial (top) and coronal (bottom) maximum intensity projections (10 mm thick) are shown to highlight differences in lesion visibility for uT_1_RESS (left) and 3D spoiled GRE (right). Small metastatic lesions are much better visualized using uT_1_RESS than with 3D spoiled GRE. The combination of twofold improved contrast and suppression of intravascular signals with uT_1_RESS is helpful to unambiguously identify small metastases.

**Fig. 4:**
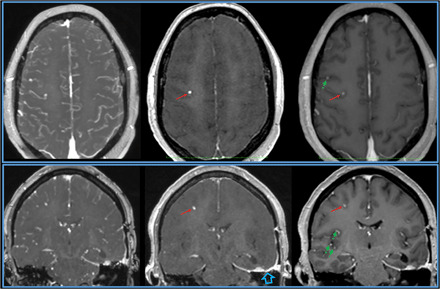
Pulse sequence comparisons in a patient with multiple breast cancer metastases to the brain. Axial (top) and coronal (bottom) 1-mm-thick axial multiplanar reformations for bT_1_RESS (left), uT_1_RESS (middle), and 3D spoiled GRE (right). With bT_1_RESS and 3D spoiled GRE, a minute metastasis (red arrow) is difficult to distinguish from enhancing blood vessels (green dashed arrows) running through the slice that have a similar appearance in cross section. However, the lesion can be unambiguously identified using uT_1_RESS because the signals from blood vessels and background tissues are well suppressed. Prominent left infratemporal contrast enhancement (blue open arrow) relates to a recent tumor resection.

**Fig. 5:**
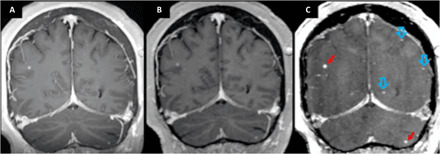
Patient with multiple metastases. (**A**) 3D inversion recovery spoiled GRE (IR-SPGRE). (**B**) 3D spoiled GRE. (**C**) uT_1_RESS. Two lesions (red arrows) are much more conspicuous with uT_1_RESS than with 3D IR-SPGRE or spoiled GRE, despite all sequences being acquired at the same spatial resolution. Multiple additional minute enhancing foci (such as the ones labeled with blue open arrows) are only visible with uT_1_RESS.

For imaging of tumors, the bland, low-signal background and sensitivity to lesion enhancement resulted in a marked improvement in the visibility of enhancing tumors compared with standard pulse sequences. With uT_1_RESS, enhancing tumors appeared particularly conspicuous against a background in which blood vessels along with nonenhancing tissues all appeared relatively dark (Fig. 3). With this method, we found that even minute metastatic tumor deposits that were difficult to distinguish from small blood vessels using three-dimensional (3D) spoiled GRE could be unambiguously identified (Figs. 4 and 5). Moreover, banding artifacts from off-resonance effects were absent with uT_1_RESS, whereas they were often seen near air–soft tissue boundaries with bT_1_RESS.

Quantitative comparisons demonstrated that bT_1_RESS significantly outperformed both 3D spoiled GRE and 3D inversion recovery (IR) spoiled GRE (IR-SPGRE) imaging with respect to vessel visibility. In 40 patients, the mean signal-to-noise ratio (SNR) of the superior sagittal sinus was 1.49-fold better with bT_1_RESS versus 3D spoiled GRE (359.43 ± 137.30 versus 241.27 ± 80.47, *P* = 3.26 × 10^−7^), while the mean vessel-to-brain contrast was improved by a factor of 2.04 (2.46 ± 0.53 versus 1.21 ± 0.33, *P* = 3.57 × 10^−8^). Both T_1_RESS versions outperformed 3D spoiled GRE and 3D IR-SPGRE with regard to tumor visibility, as gauged by tumor-to-brain contrast and contrast-to-noise ratio (CNR). For brain tumors (88 tumors in 40 patients), the mean CNR of brain tumors with respect to normal parenchyma was significantly better for bT_1_RESS versus 3D spoiled GRE (133.15 ± 100.81 versus 53.53 ± 51.15, *P* = 1.48 × 10^−14^), as was the tumor-to-brain contrast (1.25 ± 0.68 versus 0.51 ± 0.39, *P* = 7.21 × 10^−15^). Comparing uT_1_RESS with 3D spoiled GRE (84 tumors in 38 patients), the respective values for mean CNR of brain tumors were 105.80 ± 80.26 versus 54.84 ± 51.44 (*P* = 2.59 × 10^−13^), the respective values for mean tumor-to-brain contrast were 1.37 ± 0.74 versus 0.51 ± 0.39 (*P* = 1.12 × 10^−14^), and the respective values for mean tumor–to–blood vessel contrast were 1.74 ± 1.07 versus −0.29 ± 0.17 (*P* = 2.05 × 10^−15^). Comparing uT_1_RESS with 3D IR-SPGRE (45 tumors in 22 patients), the respective values for mean CNR of brain tumors were 164.37 ± 97.21 versus 100.45 ± 86.45 (*P* = 7.62 × 10^−6^), the respective values for mean tumor-to-brain contrast were 1.69 ± 0.89 versus 0.82 ± 0.55 (*P* = 5.14 × 10^−8^), and the respective values for mean tumor–to–blood vessel contrast were 2.56 ± 1.21 versus −0.15 ± 0.34 (*P* = 5.54 × 10^−9^).

**Fig. 6:**
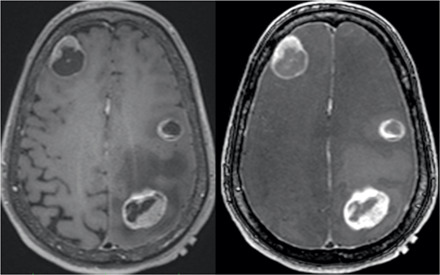
Pulse sequence comparisons in a patient with metastatic esophageal carcinoma. Comparison of T1 3D-VFA-FSE (left) with uT1RESS (right) using equal spatial resolution and scan time in a patient with multiple metastases from an esophageal carcinoma. Although both sequences effectively suppress intravascular signal, the contrast and CNR values for T1 3D-VFA-FSE were markedly inferior to uT1RESS (contrast = 0.37 versus 1.59, respectively; CNR = 51.0 versus 331.3, respectively).

uT_1_RESS also provided markedly superior tumor-to-brain contrast and CNR compared with T1 3D variable flip angle fast spin echo (3D-VFA-FSE) (Fig. 6). Comparing uT_1_RESS with T1 3D-VFA-FSE (32 tumors in 14 patients), the respective values for mean CNR of brain tumors were 180.32 ± 92.34 versus 60.34 ± 54.88 (*P* = 8.75 × 10^−7^), the respective values for mean tumor-to-brain contrast were 1.76 ± 0.76 versus 0.89 ± 0.43 (*P* = 7.95 × 10^−7^), and the respective values for mean tumor–to–blood vessel contrast were 2.58 ± 1.21 versus 11.94 ± 5.26 (*P* = 7.95 × 10^−7^).

## DISCUSSION

Several types of MRI pulse sequences are routinely used in clinical practice. The most common include spin echo and its rapid variant, fast spin echo (*5*); spoiled GRE (*6*); and balanced steady-state free precession (SSFP) (bSSFP) (*7*). For oncological applications of MRI, T1 weighting is essential to detect tumor enhancement from paramagnetic contrast agents (*8*). The spoiled GRE sequence provides excellent T1 weighting and is efficient with respect to scan time because it allows the use of short repetition times (TR). Volumetric implementations such as 3D IR-SPGRE have become essential components of cancer imaging protocols for the brain and other organ systems (*9*). However, spoiled GRE–based techniques have low SNR efficiency as they are restricted to using a low flip angle RF excitation (on the order of the Ernst angle of the tissue of interest) to avoid excessive depletion of the longitudinal magnetization (*10*). Like spoiled GRE, bSSFP also permits the use of very short TR. Unlike spoiled GRE, bSSFP can effectively use a large flip angle RF excitation due to its recycling of transverse magnetization, which results in the highest SNR efficiency of any MRI sequence (*11*).

With bSSFP, the image contrast is almost entirely determined by the ratio of the T2 and T1 relaxation times (*11*, *12*). Consequently, tissues with a high T2/T1 ratio, such as arterial blood, cerebrospinal fluid, and fat, appear bright, whereas those with a low T2/T1 ratio, such as white matter and muscle (*13*), appear dark (*14*). While this tissue contrast has proven useful for several clinical indications (*15*–*18*), its dependence on the T2/T1 ratio limits the overall clinical utility of the technique. With respect to imaging of tumors, paramagnetic contrast agents shorten both the T1 and T2 relaxation times of enhancing tissues, leaving the T2/T1 ratio (and thereby the bSSFP signal intensity) largely unchanged (*11*). Consequently, despite their potential benefits, it remains problematic to use bSSFP pulse sequences for oncological applications.

The T_1_RESS method overcomes these limitations through a redesign of the steady-state pulse sequence architecture. It introduces a flexible degree of T1 weighting into the sequence while maintaining its excellent SNR efficiency by repeatedly applying additional nonspatially selective partial saturation contrast-modifying (CMα) RF pulses throughout the duration of the echo train. The CMα RF pulses can be adjusted independently of the imaging RF pulses. The amount of T1 weighting can be changed as needed by varying the values for the CMα flip angle and TR, with larger flip angles and shorter TR resulting in more T1 weighting. For the current study, the typical values for the CMα flip angle and TR were ≈75° and ≈400 ms, respectively.

This pulse sequence redesign has two essential benefits for oncological applications: (i) It makes the T_1_RESS method highly sensitive to the T1 shortening effects of paramagnetic contrast agents, so that enhancing tumors can be well visualized, and (ii) it substantially reduces the signal intensity of nonenhancing background tissues, thereby improving the visibility of those enhancing tumors. The high degree of background signal suppression results from several factors: (i) the repeated application of the CMα RF pulses, which partially saturates the longitudinal magnetization; (ii) the low T2/T1 ratio of healthy brain tissue, which, in combination with the SSFP readout, results in a substantially diminished signal intensity relative to spoiled GRE; and (iii) magnetization transfer effects, which are much greater with T_1_RESS than spoiled GRE due to the frequent application of short-duration high flip angle imaging and CMα RF pulses (*19*).

The balanced version of T_1_RESS, which we call bT_1_RESS, demonstrated excellent tumor-to-background contrast and CNR. It also provided improved angiographic renderings of blood vessels compared with 3D spoiled GRE or IR-SPGRE. Unfortunately, having contrast-enhanced blood vessels appear bright can be a disadvantage for cancer imaging. Enhancing blood vessels distract from, and potentially may be confused with, enhancing tumors (*20*). This may be especially problematic for detecting very small tumors and leptomeningeal metastases because of the presence of bright cortical vessels nearby. Therefore, a second uT_1_RESS version was developed to provide a robust solution for this problem.

uT_1_RESS renders blood vessels dark by using a steady-state unbalanced GRE readout in which the phase-encoding gradients are rewound and the gradient along the frequency-encoding direction is unbalanced (*12*, *21*, *22*). The resultant suppression of intravascular signals is a consequence of flow- and diffusion-related phase dispersion that gradually accumulates with each sequence repetition (*23*). Because the 3D T_1_RESS acquisition uses a very large number (≈40,000) of sequence repetitions, intravascular phase dispersion is complete, resulting in marked suppression of intravascular signal regardless of vessel orientation. While unbalanced steady-state sequences are known to be more motion sensitive than bSSFP or spoiled GRE (*24*), we found that motion artifacts were generally mild or absent in our brain studies. This is likely because T_1_RESS uses a very weak dephasing gradient, which, along with the large number of sequence repetitions, ensures that the phase dispersion is gradual and consistently applied in every sequence repetition. However, this issue will require further evaluation for other regions of the body such as the abdomen, where motion is a greater concern.

A more conventional approach for dark blood imaging of brain tumors is the 3D-VFA-FSE technique, which has been shown to be highly sensitive for small brain metastases (*3*). T1 3D-VFA-FSE has been included in recent consensus recommendations for tumor imaging (*25*). While further study is needed, our results suggest that there are significant drawbacks compared with uT_1_RESS, including much lower SNR efficiency and tumor-to-background contrast, as well as longer minimum scan times. Other potential concerns include sensitivity to B1 field inhomogeneity, blurring from T2 decay if the echo train is overly long, and incomplete suppression of intravascular signals depending on the particular implementation (*26*).

This study had several limitations. Gold standard biopsies were only available in a minority of the cases. Scan times and spatial resolution varied for some of the imaging protocols, requiring normalization of the SNR. It would be preferable for future studies to keep these imaging parameters constant across imaging protocols. Residual signal was sometimes present in segments of small cortical veins. Concerns about persistent venous signal, along with other issues of potential clinical relevance, will need to be addressed in future studies.

In conclusion, T_1_RESS represents a redesign of the traditional steady-state pulse sequence architecture. This novel MRI method enables the flexible modulation of T1 weighting and provides the unique feature that intravascular signals can be toggled on and off in contrast-enhanced scans. T_1_RESS makes it possible to effectively use an MRI technique with improved SNR efficiency for cancer imaging. While this initial proof-of-concept study was not designed to determine the diagnostic accuracy of the technique, the combination of twofold improved tumor-to-background contrast and flexible control over intravascular signal has the potential to make T_1_RESS a valuable clinical tool. The improvement in contrast should facilitate the detection of cancer at an earlier stage for the brain and other organs such as the liver, breast, and prostate than is possible with current MRI techniques. In addition, the combination of high SNR efficiency, short TR, and suppression of signal from macroscopic vessels may prove advantageous for dynamic contrast-enhanced evaluation of tumor perfusion.

T_1_RESS could also prove beneficial for a range of nononcological applications. For instance, the sensitivity to contrast enhancement and suppression of intravascular signal with uT_1_RESS could prove helpful for detecting active lesions of multiple sclerosis or evaluating vessel wall inflammation, whereas the high SNR efficiency of bT_1_RESS could be leveraged to substantially reduce the contrast agent dosage or scan time needed for magnetic resonance angiography. However, further work is needed for sequence modeling, optimization, and clinical validation to realize the full potential of the technique.

## MATERIALS AND METHODS

### Experimental design

This study was approved by the hospital institutional review board with waiver of consent. Contrast-enhanced MRI of the brain was performed at 3 T (MAGNETOM Skyra and MAGNETOM Skyra^fit^, Siemens Healthcare, Erlangen, Germany) in 54 adult subjects (ages, 19 to 88 years; 27 female) with suspected or known brain tumors. For contrast-enhanced MRI of the head, gadobutrol (0.1 mmol/kg) (Bayer, Berlin, Germany) was administered intravenously, followed by standard-of-care 2D fast spin echo and, in a subset of patients, 3D IR-SPGRE. Total scan duration for these postcontrast sequences ranged from approximately 6 to 13 min. Immediately following acquisition of these sequences, three additional postcontrast scans were typically obtained, consisting of balanced and unbalanced T_1_RESS as well as an additional 3D spoiled GRE acquisition that was matched for scan time and spatial resolution with T_1_RESS.

### T_1_RESS sequence design and scan parameters

To obtain T1 weighting for a contrast-enhanced MRI scan, bSSFP traditionally incorporates a preparatory 90° saturation recovery (e.g., for first-pass contrast-enhanced perfusion imaging) or 180° IR RF pulse (e.g., for imaging of delayed myocardial enhancement). These preparatory RF pulses are followed by a waiting period of at least a few hundred milliseconds before data collection (*27*–*30*). The use of a single large flip angle preparatory RF pulse has several drawbacks: (i) It reduces the SNR; (ii) *k*-space lines acquired early in the echo train will have a markedly different amount of T1 weighting from ones acquired later on, potentially causing a loss of contrast for small lesions; and (iii) the lengthy waiting period greatly diminishes the SNR efficiency compared with an unmodified bSSFP sequence, thereby increasing scan time.

The T_1_RESS pulse sequence avoids these limitations by applying a rectangular-shaped, spatially nonselective partial saturation contrast-modifying (CMα) RF pulse at regular intervals (CMα TR) throughout the duration of a continuous 3D SSFP acquisition, without any waiting period (Fig. 1). For bT_1_RESS, the steady-state magnetization is stored along the *z* axis by a α/2(−) pulse immediately before each application of the CMα pulse, followed by a second α/2(+) pulse to restore the steady-state magnetization to its previous state (where α is the imaging flip angle). While the magnetization is stored along the *z* axis, the CMα RF pulse can be applied without disrupting the steady-state echo train. For uT_1_RESS, the CMα pulse is applied between phase-encoding segments without additional store/restore pulse pairs, while a weak gradient spoiler (20% of the default amplitude) is applied along the frequency-encoding direction between imaging RF pulses to provide a small degree of flow-related dephasing.

For both bT_1_RESS and uT_1_RESS, data are acquired using a Cartesian 3D *k*-space trajectory as a single shot along the phase-encoding direction, whereas the acquisition is segmented along the 3D partition-encoding direction. Typical sequence parameters included echo spacing of ≈2.9 ms, flip angle of the imaging RF pulse ≈50°, sampling bandwidth = 888 Hz per pixel, CMα flip angle = 75°, 7/8 slice partial Fourier, parallel acceleration factor = 2, and CMα TR ≈ 400 ms. A chemical shift-selective fat saturation RF pulse was applied along with each CMα RF pulse. Scan times were ≈1 min 45 s for two signal averages.

For T_1_RESS and 3D spoiled GRE, a 3D slab was acquired in a sagittal orientation using a rectangular-shaped, spatially nonselective RF excitation. Spatial resolution was near isotropic with reconstructed slice thickness of 0.45 mm and in-plane spatial resolution of 0.5 mm. The 3D spoiled GRE acquisition used an echo spacing of 5.5 ms, flip angle of 11°, and sampling bandwidth of 395 Hz per pixel. Scan time for 3D spoiled GRE was ≈1 min 49 s. Standard-of-care 3D IR-SPGRE was acquired in an axial orientation with 1-mm^3^ isotropic spatial resolution, TR = 1900 ms, TI = 900 ms, TE = 2.4 ms, parallel acceleration factor = 2, sampling bandwidth = 250 Hz per pixel, and scan time = 4 min 30 s.

In 14 patients, both T1 3D-VFA-FSE and uT_1_RESS were acquired using approximately 1-mm^3^ isotropic spatial resolution. T1 3D-VFA-FSE was acquired using default parameters (e.g., TR = 700 ms, echo train length = 44, sampling bandwidth = 435 Hz per pixel), except that the slice parallel acceleration factor was increased from 2 to 4 to reduce the scan time to 3.5 min. The number of signal averages was increased from 2 to 4 for uT1RESS to match this scan time.

### Signal measurement and statistical analysis

Region-of-interest signal measurements were obtained in brain lesions, in nearby normal brain tissue, in air, and in the superior sagittal sinus. Given that SNR per voxel was well above the Rose threshold of 4 to distinguish image features with certainty, it is unlikely that image noise plays much role in lesion visibility for the MRI scans used in this study. Therefore, we used a calculation analogous to Weber contrast, computed as (SI_tumor_ − SI_normal_)/SI_normal_, as the primary metric for lesion visibility (*31*). In addition, the CNR was used as a secondary metric for lesion visibility, calculated as 0.655*(SI_tumor_ − SI_normal_)/SD_air_. SI_tumor_ and SI_normal_ are the mean signal intensities of the tumor and normal-appearing adjacent normal brain tissue, and SD_air_ is the standard deviation within air above the head. To normalize for the 2.57-fold longer scan time of the 3D IR-SPGRE sequence and the 2-fold longer scan time of T1 3D-VFA-FSE and 4-average uT_1_RESS, the CNR was multiplied by 1/√2.57 and 1/√2, respectively. Quantitative measures were compared using Wilcoxon signed-rank tests. Statistical comparisons were done using the SciPy computing library (version 1.4.1, https://scipy.org/scipylib/). Data were presented as mean ± SD.

## Supplementary Material

http://advances.sciencemag.org/cgi/content/full/6/44/eabd1635/DC1

Movie S1

Adobe PDF - abd1635_SM.pdf

Twofold improved tumor-to-brain contrast using a novel T1 relaxation-enhanced steady-state (T1RES) MRI technique

## SUPPLEMENTARY MATERIALS

Supplementary material for this article is available at http://advances.sciencemag.org/cgi/content/full/6/44/eabd1635/DC1

## Appendix

View/request a protocol for this paper from *Bio-protocol*.
