# Supplementary material for: Twofold improved tumor-to-brain contrast using a novel T1 relaxation-enhanced steady-state (T1RESS) MRI technique
Source: Sci Adv. 2020 Oct 28;6(44):eabd1635. doi: 10.1126/sciadv.abd1635 (PMC7608787; doi:10.1126/sciadv.abd1635)
Supplement: http://advances.sciencemag.org/cgi/content/full/6/44/eabd1635/DC1 [file supp_6_44_eabd1635__index.html]

Science Advances | Science AdvancesAAASSearchScience AdvancesMenu

## Supplementary Materials

# Twofold improved tumor-to-brain contrast using a novel T1 relaxation-enhanced steady-state (T1RESS) MRI technique

R. Edelman, N. Leloudas, J. Pang, J. Bailes, R. Merrell, I. Koktzoglou

Download Supplement

**The PDF file includes:**

- Legend for movie S1

**Other Supplementary Material for this manuscript includes the following:**

- Movie S1

**Files in this Data Supplement:**

- Adobe PDF - abd1635\_SM.pdf
