# Supplementary material for: Twofold improved tumor-to-brain contrast using a novel T1 relaxation-enhanced steady-state (T1RESS) MRI technique
Source: Sci Adv. 2020 Oct 28;6(44):eabd1635. doi: 10.1126/sciadv.abd1635 (PMC7608787; doi:10.1126/sciadv.abd1635)
Supplement: http://advances.sciencemag.org/cgi/content/full/6/44/eabd1635/DC1 [file supp_6_44_eabd1635__1.pdf]

Science Advances
AAAS

advances.sciencemag.org/cgi/content/full/6/44/eabd1635/DC1

Supplementary Materials for

Twofold improved tumor-to-brain contrast using a novel T1 relaxation-enhanced steady-state (T_1RESS) MRI technique

R. Edelman*, N. Leloudas, J. Pang, J. Bailes, R. Merrell, I. Koktzoglou

*Corresponding author. Email: redelman999@gmail.com

Published 28 October 2020, *Sci. Adv.* **6**, eabd1635 (2020)
DOI: 10.1126/sciadv.abd1635

The PDF file includes:

Legend for movie S1

Other Supplementary Material for this manuscript includes the following:

(available at advances.sciencemag.org/cgi/content/full/6/44/eabd1635/DC1)

Movie S1

Supplementary Materials:

Movie S1. 128-mm thick projection angiograms created from post-contrast 3D acquisitions. bT_1RESS (right) shows markedly improved vascular detail compared with 3D spoiled GRE (left) due to the excellent vascular SNR and improved background tissue suppression.
